# Biodiversity in Tomatoes: Is It Reflected in Nutrient Density and Nutritional Yields Under Organic Outdoor Production?

**DOI:** 10.3389/fpls.2020.589692

**Published:** 2020-11-24

**Authors:** Cut Erika, Stefanie Griebel, Marcel Naumann, Elke Pawelzik

**Affiliations:** ^1^Division Quality of Plant Products, Department of Crop Sciences, Faculty of Agricultural Sciences, University of Göttingen, Göttingen, Germany; ^2^Division Plant Breeding Methodology, Department of Crop Sciences, Faculty of Agricultural Sciences, University of Göttingen, Göttingen, Germany

**Keywords:** tomato, biodiversity, nutrients, nutritional yield, dietary reference intake, genotypic stability

## Abstract

In many regions of the world, human nutrition is still characterized by an insufficient intake of essential nutrients like minerals such as iron (Fe) and zinc (Zn). In view of decreasing resources and a growing world population, the efficiency and the sustainability of cultivation systems should be considered not only in terms of crop yield and profit margin but also in terms of the yield of essential nutrients. Tomatoes are the most consumed vegetable in the world. Organic outdoor tomato cultivation is generally characterized by a higher diversity of varieties and lower fertilization input compared to conventional production. A 2-year field experiment with a set of 20 cultivars was performed to evaluate their variation regarding fruit mineral concentrations [potassium (K), calcium (Ca), magnesium (Mg), phosphorous (P), Fe, and Zn], their contribution to the dietary reference intake (DRI), and the nutritional yields (adults ha^–1^ year^–1^). Results show that mineral concentrations differed significantly by cultivar and by year. However, even though significant genotype-by-year effects appear, several cultivars exhibit high genotype stability across years for the single traits studied. Taking this together with medium-to-high heritability, genetics strongly controls most studied traits. Among the cultivars, the contribution of 100 g fresh fruits varied from 4.5 to 7.7% for K, 0.8 to 1.8% for Ca, 2.3 to 4.4% for Mg, 3 to 6.6% for P, 3.1 to 6.9% for Fe, and 1.9 to 4.2% for Zn to meet daily requirements. Based on average fruit yields per hectare, the cultivars varied with regard to the nutritional yields for all the studied minerals, but most strongly for Fe (44–120 adults ha^–1^ year^–1^) and Zn (22–84 adults ha^–1^ year^–1^). In terms of contribution to the DRI and nutritional yield for Fe, the cocktail cultivar “Bartelly F1” produced the highest results, while for Zn the salad cultivar “Bocati F1” showed the highest values. Our results show that the targeted use of tomato biodiversity in organic outdoor production can be suitable to achieve high fruit yields as well as to produce high nutritional yields per unit area, thus contributing to more effective land use and improved food security. These findings also provide valuable insights for tomato breeders to improve the tomato fruit quality while maintaining yield.

## Introduction

The demand-oriented nutrition of a growing world population with a simultaneous reduction in the area of arable land per inhabitant and the shortage of other resources is one of the greatest challenges in the coming years (Springmann et al., 2018). In the past, increasing yields through improved nutrient utilization (e.g., Branca et al., 2013), crop protection, and cultivation techniques (Pretty and Bharucha, 2014) as well as breeding (Tieman et al., 2017) have been the main focus. Intensive land use has contributed to the increase in many environmental problems, such as rising emissions ((Foley et al., 2011) as well as the contamination of soil and water with residues (Ridoutt et al., 2017). In global terms, there is still no substantial improvement in the nutritional situation, but rather many forms of malnutrition: on the one hand malnutrition due to a deficiency in micronutrients and obesity and diet-related diseases on the other hand (Fears et al., 2019). A major cause of this is the one-sided focus on the production of nutritional energy and protein, which has led to a steady decrease in the number of cultivated crops (FAOSTAT, 2019) and varieties within crops (Martin et al., 2019). Using FAO data, Martin et al. (2019) have compiled the changes in the number of cultivars for human nutrition over the period 1961–2014. In Western Europe, the number of tomato cultivars decreased by 78% and of potato cultivars by 77%, while for wheat they increased by 43%. Continuing concerns about global food security and food quality, particularly for increased nutrient content, need to be improved with reduced inputs (Tester and Langridge, 2010). While the biodiversity on and around agricultural land may be higher, organic agriculture may require more land than conventional to produce the same yield (Schrama et al., 2018).

Based on the increasing recognition of the link between sustainable land use and food security, more comprehensive evaluation parameters are necessary. The aim is not to consider the crop yield per hectare or calorie uptake as assessment criteria but the nutrients produced per hectare and the number of people who can be fed for a full year from the nutrients produced per hectare (De Fries et al., 2015). Nutritional yield (NY) was introduced, in the form described here, by De Fries et al. (2015) and is calculated on the basis of nutrient yield per hectare and the recommended food intake. This also allows the nutritional quality of the cultivated species to be considered. There are some studies on the NY of minerals in cereals (De Fries et al., 2015, 2016; Moreira-Ascarrunz et al., 2016), legumes (Graham et al., 2018), or of the constituents in several types of plant and animal foods (De Ruiter et al., 2018). Individual vegetable species, which are frequently consumed due to their easy availability and popularity, have not yet been examined in detail under the aspect of NY.

Globally, tomato (*Solanum lycopersicum* L.) is a major cultivated and consumed fruit vegetable with per capita consumption of either fresh or processed type of about 21 kg in 2017 or around 19% of the total vegetable consumption per year (FAOSTAT, 2020). It is a rich source of macro- and micronutrients (Bauchet and Causse, 2012), vitamins, and phytochemicals for human diet (Viskelis et al., 2015; Uluisik et al., 2016; Tieman et al., 2017). In Germany, tomato production covered, in 2019, an area of 385.63 hectares, and organic production accounted for 21% of those cultivated (Statistisches Bundesamt, 2020). However, the quantities are expected to increase (Zörb et al., 2020) due to consumers’ growing attention to organic systems for reasons of health, safety, and environmental benefits (Ordóñez-Santos et al., 2011; Johansson et al., 2014).

Tomatoes have been cultivated for about 400 years, and substantial breeding activities have been implemented for only eight decades. So far, more than 10,000 tomato cultivars have been developed (Bhattarai et al., 2018). Beginning in the 20th century, through intensive breeding activities, scientists and breeders worldwide created a wide array of morphologically different cultivars from the single species *S. lycopersicum* to modern tomato varieties with high variation in fruit weight, fruit size and shapes, and colors (Bai and Lindhout, 2007).

The focus of modern breeding programs for fresh market use of tomato have usually laid emphasis on resistance, yield, and quality attributes such as firmness, color, texture, and traits related to fruit appearance (Foolad, 2007) rather than on sustainable production and nutritional quality (Mata-Nicolás et al., 2020).

In the present study, the approach of NY was applied to assess the trade-off between yields and nutrients, and the potential of tomato cultivars differing in fruit size and color to meet the human nutritional requirements of mineral nutrients. The objectives of the present work were (i) to estimate the production potential and contribution of the cultivars differing in their fruit type to meet human dietary needs for several nutrients, (ii) to assess the NY of the cultivars grown in a 2-year field experiment for selected macro- and micronutrients, and (iii) to evaluate the heritability of traits and the genotypic value and stability of the cultivars. The macronutrients K, Ca, Mg, and P are the major elements in the tomato fruit (Hernández Suárez et al., 2007), and Fe and Zn, as indispensable micronutrients, are involved in various metabolic processes (Costa et al., 2011; Jha and Warkentin, 2020).

The example of tomato will be used to investigate how a diversity of varieties is reflected in mineral composition and how this is expressed in NY. Selecting a cultivar with a greater NY will increase the contribution of tomato for human diet, with efficient use of land while still providing adequate amounts of nutrients and achieving agricultural sustainability.

## Materials and Methods

### Plant Material and Cultural Practice

Twenty indeterminate tomato cultivars (Table 1) were grown in organic low-input conditions in the field under a well-ventilated rainout shelter. The study was carried out during summer 2015 and 2016 at the experimental research station of the University of Göttingen, Germany. The 20 cultivars included 12 cocktail and 8 salad cultivars and were grouped within their fruit type on the basis of the average fruit weight. The main determinant of the tomato type definition is not well specified in the scientific literature, although the traits related to fruit size and shape seem to be the most important factors for the fruit type classification (Lázaro, 2018). Cocktail tomatoes were described as small-sized type of tomatoes—they are hybrids of cherry tomatoes with normal-sized cultivars, e.g., salad tomatoes (Kagan-Zur and Mizrahi, 1993). In this study, cultivars with fruit weight less than 52 g were classified as cocktail tomatoes, while cultivars with higher fruit weight were categorized as salad tomatoes. The main selection criteria for these cultivars were the yield and the parameters that determine the taste and the aroma formation.

**TABLE 1:** Overview of the tested cultivars.

Cultivar	Fruit type^a^	Fruit color	Fruit weight^b^ (g)	
			2015	2016
Goldita	Cocktail	Orange	15.6	17.2
Supersweet 100 F1	Cocktail	Red	15.7	13.7
Resi	Cocktail	Red	17.3	20.4
Bartelly F1	Cocktail	Red	18.4	14.9
Benarys Gartenfreude	Cocktail	Red	18.5	19.1
Primavera	Cocktail	Red	21.3	21.6
Black Cherry	Cocktail	Red-brown	23.0	25.2
Sakura F1	Cocktail	Red	23.7	24.1
Primabella	Cocktail	Red	28.1	27.8
Tastery F1	Cocktail	Red	33.5	32.1
Annamay F1	Cocktail	Red	46.0	48.2
Amoroso F1	Cocktail	Red	50.8	47.4
Campari F1	Salad	Red	63.3	62.5
Auriga	Salad	Orange	71.5	75.1
Harzfeuer F1	Salad	Red	76.4	72.1
Roterno F1	Salad	Red	106.7	104.6
Lyterno F1	Salad	Red	115.9	115
Bocati F1	Salad	Red	124.4	114.4
Cappricia F1	Salad	Red	131.5	124.1
Green Zebra	Salad	Green-yellow	153.0	136.4

*^a^Fruit type is defined by an average fruit weight of <52 g for cocktail cultivars and >52 g for salad cultivars. ^b^Fruit weight was calculated as the average single fruit weight of each cultivar for each year derived from eight biological replicates.*

The seeds were germinated in the substrate “Anzuchtsubstrat Organisch” (Kleeschulte GmbH & Co. KG, Germany) on March 30, and the seedlings were potted after 20 days in the substrate “Hawita Fruhstorfer Bio-Aussaat-und Kräutererde” (HAWITA-Gruppe GmbH, Germany). The seedlings were maintained under greenhouse conditions (20°C day, 18°C night, 16/8 h) before being transplanted to the field. The field trials were established in a randomized complete block design with eight replications. The cultivars were assessed with one plant per plot in 2015 and two plants per plot in 2016. All the plants were cultivated and spaced at 50 cm within the row and spaced at 100 cm at a population of two plants per square meter. In both years, the plants were grown under organic low-input conditions without fertilization and moderate irrigation. Further information on the growth conditions in the field is described in Table 2.

**TABLE 2:** Information about field location and cultural practices in 2015 and 2016.

	2015	2016
Location	51°30′17.6″ N, 9°55′16.2″ E	
District/region	Göttingen, Lower Saxony	
Experimental design	RCBD, eight replicates	
Soil type/properties	Fluventic Eutrochrept soil	
Average temperature (°C)^a^	19 ± 4.6	19 ± 7.0
Average relative humidity (%)^a^	75 ± 10.7	75 ± 18.9
Pre-crop	Faba bean	Winter wheat
	(*Vicia faba*)	(*Triticum aestivum*)
Soil pH	7	6.9
Humus content (%)	1.89	1.87
Phosphorus (mg 100 g^–1^ soil)	7	6
Magnesium (mg 100 g^–1^ soil)	4	7
Potassium (mg 100 g^–1^ soil)	9	9
Planting date	May 20	May 20
Planting arrangement	One plant per plot	Two plants per plot
Plant density (plants ha^–1^)	20.000	
Weed control	Space between rows were covered with plastic layers	
Main shoot pruning	Weekly, hand pruning	
Frequency/total amount of irrigation	Weekly/150 l m^–2^	

*^a^Data were recorded every 30 min from planting to final harvest using an EBI 20-TH Data Logger (ebro Electronic GmbH & Co. KG). RCBD, randomized complete block design.*

### Determination of Fruit Yield

At full maturity, the fruits were harvested at 2-week intervals, starting from 9 weeks after planting (WAP) in 2015 and 8 WAP in 2016. All the plants of each block from eight biological replications were harvested and weighed by pooled replications to obtain the total yield (kg plant^–1^) of fully ripe healthy fruits. Next, the mean fruit weight for each of the 20 evaluated plants was used to calculate the fruit yield per hectare and converted to fruit yield in tons per hectare (tons ha^–1^).

### Determination of Minerals in Fruits

Samples from two harvest dates for both years (2015: 13 WAP and 18 WAP; 2016: 14 WAP and 19 WAP) were used for the determination of mineral concentration. At harvest, the fully ripe fruits of eight biological replicates were combined into four pooled replicates for further analysis. Each 10 fruits of cocktail cultivars and 3 fruits of salad cultivars were cut into wedges. The fruits were freeze-dried (EPSILON 2-40, Christ, Osterode am Harz, Germany) for 4 days and were later ground by using a ball mill (45 s at 30 Hz; Retsch, model: MM 400, Germany) to get fine powder of up to 0.5 mm in particle size. The mineral concentration was determined according to Koch et al. (2019), with minor changes. In total, 100 mg of the ground fruit material was weighed in a Teflon vessel and digested in 4 ml of 65% (v/v) concentrated HNO_3_ and 2 ml of 30% concentrated H_2_O_2_ (Carl Roth GmbH & Co. KG, Germany) for 75 min at 200°C and 40 bar in a microwave oven (ETHOS Professional Microwave System, MLS GmbH, Germany). After microwave digestion, the samples were transferred to volumetric flasks and filled up with distilled water to a total volume of 25 ml. The concentrations of macronutrients (Ca, K, Mg, and P) and micronutrients (Fe and Zn) were analyzed using inductively coupled plasma-optical emission spectrometry (Vista-RL, Variance Inc., Palo Alto, CA, United States). The concentrations of minerals were expressed in mg/100 g fresh weight (FW).

### Calculations and Statistical Analysis

In this study, the fraction of the dietary reference intake (DRI) was defined as the percentage of the nutrient requirement provided by 100 g of tomatoes based on the assessed mineral concentration for each cultivar. The DRIs are derived from the values released by the Food and Nutrition Board of the Institute of Medicine (IOM) and have been widely used in recommending quantitative estimates of nutrient intakes for the United States and Canadian populations (Institute of Medicine, 2006). The fraction of DRI is calculated as grams of a mineral divided by the DRI values. The values for DRI*i* are taken either from the recommended daily allowance or the adequate intake for average adult males and females (not pregnant or lactating) aged between 19 and 50 years (Supplementary Table 1). The fraction of DRI of each mineral *i* from a tomato cultivar *j* was calculated as:

(1) $$\mathrm{fraction}\mathrm{DRI}=\frac{{\text{g}}_{i}/100{\text{g}}_{j}}{{\text{DRI}}_{i}}$$

where g_*i*_/100g_*j*_ is the value for grams of a mineral *i* in 100 g of a tomato cultivar *j*.

The NY weighs the conventional yield measure (ton ha^–1^) by its nutritional content, and this value is divided by the dietary requirement necessary for 1 year (De Fries et al., 2016). This metric estimates the number of adults (average for male and female between 19 and 50 years old) who can fulfill 100% of their DRI for selected nutrients from 1 ha for 1 year (De Fries et al., 2016).

Therefore, the NY of a mineral *i* from a tomato cultivar *j* was calculated as:

(2) $$NY_{ij}=\mathrm{fraction}\mathrm{of}{\mathrm{DRI}}_{i}/100{\text{g}}_{j}\times {\text{tons}}_{j}/\text{ha}/\text{year}\times 10^{4}/365$$

where the fraction of DRI_*i*_/100g_*j*_ = g_*i*_/100g_*j*_/DRI_*i*_

The fraction of DRI of nutrient *i*, provided by 100 g of cultivar *j*, was calculated as grams of each mineral *i* in 100 g of each tomato cultivar *j* divided by the daily dietary requirement for the respective mineral *i* (*g_*i*_/*100 g*_*j*_*)/DRI*_*i*_*. For example, a tomato’s NY for K is derived from the fraction of DRI for K supplied by 100 g of tomato (which is grams of K in 100 g of tomatoes divided by the daily dietary requirement for K) multiplied by the yield for the respective cultivar.

Four replications of each cultivar were maintained in each year of cultivation. The data were presented as means of each cultivar over two cultivation years. The effect of cultivar and year on the mineral concentration was evaluated by means of analysis of variance. The variances between the cultivar and the treatment means for all the traits were separated by pairwise means comparisons with Tukey’s test at *p* < 0.05 by using the STATISTIX statistical software (Version 8.0).

The heritability and the genotypic values were calculated for single years based on a linear, fully randomized model using the R package lme4 (Bates et al., 2015). Heritability (*H*^2^) was calculated as broad sense heritability for single years:

(3) $$H^{2}=\frac{\delta^{2}\text{g}}{\delta^{2}\text{g}+\delta^{2}\text{e}}$$

Where δ^2^g was the estimated genetic variance and δ^2^e was the residual variance. Best linear unbiased predictors (BLUPs) extracted from the model were reported as genotypic values. The genotypic values and the grand mean were summed up to obtain the predicted means of a trait for a single cultivar. Pearson and Spearman’s rank correlations were calculated between each year’s BLUPs of a single trait.

## Results

### Fruit Mineral Concentrations and Their Contribution to the Dietary Reference

The concentrations of macronutrients in the fruits of the cocktail cultivars ranged on the basis of FW for K between 211 mg 100 g^–1^ (“Roterno F1”) and 361 mg 100 g^–1^ (“Supersweet 100 F1”), for Ca between 7.9 mg 100 g^–1^ (“Auriga”) and 17.7 mg 100 g^–1^ (“Resi”), for Mg between 8.4 mg 100 g^–1^ (“Cappricia F1”) and 16 mg 100 g^–1^ (“Supersweet 100 F1”), and for P between 20.9 mg 100 g^–1^ (“Roterno F1”) and 46.1 mg 100 g^–1^ (“Resi”). The contents for Zn ranged between 0.18 mg 100 g^–1^ (“Cappricia F1”) and 0.40 mg 100 g^–1^ (“Resi”), while the Fe content varied from 0.41 mg 100 g^–1^ (“Cappricia F1”) to 0.90 mg 100 g^–1^ (“Resi”). The traits with a higher coefficient of variation (CV) were the concentrations of Zn (43.9%), Fe (37.4%), and Ca (23.2%), while the lowest CV was found in the concentrations of K, Mg, and P. Considering the fruit type, i.e., cocktail versus salad, it was found that the cocktail cultivars tended to have higher concentrations of all the nutrients studied. Based on the averaged sums of all nutrients, the cocktail cultivars contained about 20% higher contents than the salad cultivars (Table 3).

**TABLE 3:** Fruit concentrations of K, Ca, Mg, P, Fe, and Zn (mg 100 g^–1^ FW) by cultivar.

Cultivar	Concentration (mg 100 g^–1^ FW)					
	K	Ca	Mg	P	Fe	Zn
*Goldita*	312 ± 32	16.1 ± 4.2	14.0 ± 2.0	32.3 ± 6.4	0.76 ± 0.37	0.38 ± 0.25
*Supersweet 100 F1*	361 ± 45	10.2 ± 2.1	16.0 ± 1.2	40.8 ± 4.6	0.79 ± 0.27	0.39 ± 0.15
*Resi*	340 ± 63	17.7 ± 4.0	15.8 ± 3.7	46.1 ± 8.5	0.90 ± 0.15	0.40 ± 0.16
*Bartelly F1*	323 ± 37	14.6 ± 3.3	14.2 ± 1.5	35.8 ± 4.8	0.69 ± 0.21	0.34 ± 0.08
*Benarys Gartenfreude*	336 ± 64	8.8 ± 2.5	13.2 ± 3.5	36.1 ± 7.7	0.82 ± 0.54	0.37 ± 0.20
*Primavera*	278 ± 27	12.2 ± 2.2	11.2 ± 1.3	29.9 ± 3.2	0.73 ± 0.30	0.39 ± 0.37
*Black Cherry*	263 ± 59	11.1 ± 2.9	12.4 ± 2.7	27.7 ± 6.3	0.68 ± 0.22	0.31 ± 0.08
*Sakura F1*	305 ± 41	12.1 ± 3.0	13.6 ± 2.1	31.8 ± 3.5	0.63 ± 0.25	0.32 ± 0.08
*Primabella*	324 ± 39	11.5 ± 3.6	14.2 ± 1.7	35.8 ± 5.1	0.78 ± 0.19	0.33 ± 0.14
*Tastery F1*	271 ± 28	11.9 ± 2.8	12.2 ± 1.4	29.4 ± 4.0	0.62 ± 0.13	0.30 ± 0.13
*Annamay F1*	299 ± 29	13.3 ± 2.4	13.5 ± 1.3	29.3 ± 3.1	0.62 ± 0.15	0.35 ± 0.20
*Amoroso F1*	244 ± 23	12.4 ± 2.0	10.6 ± 1.1	27.7 ± 2.3	0.61 ± 0.21	0.28 ± 0.10
Campari F1	278 ± 34	12.0 ± 2.3	12.0 ± 1.4	29.2 ± 4.1	0.60 ± 0.43	0.26 ± 0.12
Auriga	282 ± 34	7.9 ± 1.3	13.2 ± 1.3	27.3 ± 3.2	0.49 ± 0.16	0.22 ± 0.04
Harzfeuer F1	294 ± 34	11.1 ± 3.0	10.7 ± 1.1	26.1 ± 3.2	0.54 ± 0.23	0.24 ± 0.06
Roterno F1	211 ± 58	12.4 ± 4.0	8.6 ± 2.4	20.9 ± 6.3	0.42 ± 0.12	0.19 ± 0.04
Lyterno F1	232 ± 23	12.8 ± 3.6	9.3 ± 0.6	24.3 ± 3.4	0.44 ± 0.12	0.21 ± 0.06
Bocati F1	217 ± 35	11.9 ± 2.7	9.1 ± 1.4	21.6 ± 3.6	0.43 ± 0.15	0.19 ± 0.05
Cappricia F1	236 ± 41	13.9 ± 3.1	8.4 ± 1.0	23.3 ± 4.8	0.41 ± 0.17	0.18 ± 0.07
Green Zebra	290 ± 34	12.3 ± 2.1	14.1 ± 1.5	29.4 ± 3.4	0.55 ± 0.17	0.24 ± 0.08
Mean	285.2	12.3	12.3	30.3	0.6	0.3
CV (%)	12.9	23.2	13.0	14.8	37.4	43.9
HSD	46.20	3.57	2.00	5.61	0.29	0.16
Year						
2015	274 ± 43.4	12.0 ± 3.8	11.7 ± 2.2	29.6 ± 6.4	0.53 ± 0.26	0.34 ± 0.20
2016	296 ± 66.2	12.6 ± 3.4	13.0 ± 3.3	31.0 ± 8.8	0.69 ± 0.28	0.25 ± 0.08
Source of variation						
Cultivar	***	***	***	***	***	***
Year	***	ns	***	**	***	***
Cultivar × year	***	*	***	***	*	*

The cultivars are arranged according to their average single fruit weight (see Table 1); cocktail cultivars are indicated in italics; the others are salad cultivars. Mean values are given for each of the 20 cultivars as mean from both years and from each year ± standard deviation. CV, coefficient of variation; HSD, honestly significant difference at p ≤ 0.05; ns, not significant. *p ≤ 0.05, **p ≤ 0.01, ***p ≤ 0.001.

Overall, the nutrient densities of the determined minerals were lower in 2015 than in 2016, except for Zn. The cultivar had a very strong effect on the variance of all nutrient concentrations, followed by the effect of year—except for Ca. In general, the cultivar was dependent on the year of cultivation at different significance levels.

Over all cultivars and years, the study showed that tomatoes can contribute for K with 6.1% at most to the DRI of this nutrient, and it varied from 4.5% (“Roterno F1”) to 7.7% (“Supersweet 100 F1”). The cultivars can meet the need of the DRI of Ca on average with 1.2% within a range from 0.8% (“Auriga”) to 1.8% (“Resi”). Fresh consumption of 100 g of the studied cultivars will provide Mg from 2.3% (“Cappricia F1”) to 4.4% (“Resi” and “Supersweet 100 F1”) and P from 3.0% (“Roterno F1”) to 6.6% (“Resi”) of the DRI (Table 4). Based on the presented data, the consumption of 100 g tomato fruits will provide on average 3.4 and 4.3% of the DRI of Mg and P, respectively. When the fruit type was considered, it was shown that cocktail cultivars contributed more to the DRI than salad cultivars; i.e., “Resi” contributed at most to the Ca, Mg, and P requirements in human nutrition, while “Supersweet 100 F1” was the highest contributor for providing K and Mg.

**TABLE 4:** Contribution of cultivars to the dietary reference intake (DRI) for K, Ca, Mg, P, Fe, and Zn.

Cultivar	DRI (%)					
	K	Ca	Mg	P	Fe	Zn
*Goldita*	6.6 ± 0.7	1.6 ± 0.4	3.9 ± 0.5	4.6 ± 0.9	5.9 ± 2.8	4.0 ± 2.6
*Supersweet 100 F1*	7.7 ± 1.0	1.0 ± 0.2	4.4 ± 0.3	5.8 ± 0.7	6.1 ± 2.1	4.1 ± 1.5
*Resi*	7.2 ± 1.3	1.8 ± 0.4	4.4 ± 1.0	6.6 ± 1.2	6.9 ± 1.2	4.2 ± 1.6
*Bartelly F1*	6.9 ± 0.8	1.5 ± 0.3	3.9 ± 0.4	5.1 ± 0.7	5.3 ± 1.6	3.6 ± 0.8
*Benarys Gartenfreude*	7.2 ± 1.4	0.9 ± 0.2	3.6 ± 1.0	5.2 ± 1.1	6.3 ± 4.1	3.9 ± 2.2
*Primavera*	5.9 ± 0.6	1.2 ± 0.2	3.1 ± 0.4	4.3 ± 0.5	5.6 ± 2.3	3.1 ± 0.6
*Black Cherry*	5.6 ± 1.3	1.1 ± 0.3	3.4 ± 0.7	4.0 ± 0.9	5.2 ± 1.7	3.2 ± 0.9
*Sakura F1*	6.5 ± 0.9	1.2 ± 0.3	3.7 ± 0.6	4.5 ± 0.5	5.4 ± 2.8	3.3 ± 0.9
*Primabella*	6.9 ± 0.8	1.1 ± 0.4	3.9 ± 0.5	5.1 ± 0.7	6.0 ± 1.5	3.5 ± 1.4
*Tastery F1*	5.8 ± 0.6	1.2 ± 0.3	3.4 ± 0.4	4.2 ± 0.6	4.7 ± 1.0	3.2 ± 1.3
*Annamay F1*	6.4 ± 0.6	1.3 ± 0.2	3.7 ± 0.4	4.2 ± 0.5	4.8 ± 1.2	3.6 ± 2.1
*Amoroso F1*	5.2 ± 0.5	1.2 ± 0.2	2.9 ± 0.3	4.0 ± 0.3	4.7 ± 1.6	3.0 ± 1.0
Campari F1	5.9 ± 0.7	1.2 ± 0.2	3.3 ± 0.4	4.2 ± 0.6	4.6 ± 3.3	2.7 ± 1.3
Auriga	6.0 ± 0.7	0.8 ± 0.1	3.6 ± 0.4	3.9 ± 0.5	3.8 ± 1.2	2.3 ± 0.4
Harzfeuer F1	6.3 ± 0.7	1.1 ± 0.3	3.0 ± 0.3	3.7 ± 0.5	4.1 ± 1.8	2.5 ± 0.7
Roterno F1	4.5 ± 1.2	1.2 ± 0.4	2.4 ± 0.7	3.0 ± 0.9	3.2 ± 1.0	2.0 ± 0.5
Lyterno F1	4.9 ± 0.5	1.3 ± 0.4	2.6 ± 0.2	3.5 ± 0.5	3.4 ± 0.9	2.2 ± 0.6
Bocati F1	4.6 ± 0.7	1.2 ± 0.3	2.5 ± 0.4	3.1 ± 0.5	3.3 ± 1.2	1.9 ± 0.6
Cappricia F1	5.0 ± 0.9	1.4 ± 0.3	2.3 ± 0.3	3.3 ± 0.7	3.1 ± 1.3	1.9 ± 0.7
Green Zebra	6.2 ± 0.7	1.2 ± 0.2	3.9 ± 0.4	4.2 ± 0.5	4.2 ± 1.3	2.5 ± 0.8
Mean	6.1	1.2	3.4	4.3	4.8	3.1
CV	12.9	23.2	13.0	14.8	38.8	35.3
HSD	0.98	0.36	0.55	0.80	2.94	1.37
Year						
2015	5.8 ± 0.9	1.2 ± 0.4	3.2 ± 0.6	4.2 ± 0.9	4.18 ± 2.10	3.48 ± 1.77
2016	6.3 ± 1.4	1.3 ± 0.3	3.6 ± 0.9	4.4 ± 1.3	5.35 ± 2.17	2.61 ± 0.84

*The DRI is the fraction (%) of mineral requirement provided by 100 g fresh tomato fruit based on the mineral concentration for each cultivar (see Table 3). The cultivars are arranged according to average single fruit weight (see Table 1); cocktail cultivars are indicated in italics; the others are salad cultivars. Mean values are given for each of the 20 cultivars as mean from both years and from each year ± standard deviation. CV, coefficient of variation; HSD, honestly significant difference at p ≤ 0.05.*

The studied cultivars can contribute with an average of 4.8% for Fe and 3.1% for Zn to the DRI. Among the cultivars, the fraction of DRI for Fe ranged between 3.1% in “Cappricia F1” and 6.9% in “Resi.” For Zn, the lowest contribution to the DRI with 1.9% was found in “Cappricia F1” and the highest with 4.2% in “Resi” (Table 4).

### Fruit Yield and Its Variation in Nutritional Yield Across Years

The cultivars showed a large variation in average fruit yield and NY (Table 5). The cultivar with the highest average fruit yield across the 2 years was the salad cultivar “Roterno F1” (116 tons ha^–1^), followed by other salad cultivars “Lyterno F1” (110.3 tons ha^–1^), “Bocati F1” (112.7 tons ha^–1^), and “Cappricia F1” (112.1 tons ha^–1^). The lowest average fruit yield showed the cocktail cultivar “Resi” (22.7 tons ha^–1^), which significantly differed from all the other investigated cultivars. On average, the cultivars produced 70.4 tons ha^–1^ in 2015 and 77.4 tons ha^–1^ of ripe fruits in 2016. Although in both cultivation years the mean temperature (19°C) and relative humidity (75%) in the field were similar (Table 2), low temperatures (less than 10°C) occurred at early 18th WAP in 2015 and persisted for several days, while in 2016 temperatures below 10°C were only observed later than 18th WAP (Supplementary Figures 1.1 and 1.2).

**TABLE 5:** Variation of fruit yield and nutritional yield (adults year^–1^ ha^–1^) for K, Ca, Mg, P, Fe, and Zn by cultivar.

Cultivar	Fruit yield (tons ha^–1^)	Nutritional yield (adults year^–1^ ha^–1^)					
		K	Ca	Mg	P	Fe	Zn
*Goldita*	42.5 ± 5.6	78 ± 16.6	19 ± 6.0	45 ± 9.0	54 ± 14.1	71 ± 37.4	44 ± 15.1
*Supersweet 100 F1*	58.0 ± 7.9	123 ± 27.3	16 ± 3.7	70 ± 13.4	93 ± 17.3	100 ± 38.9	55 ± 26.4
*Resi*	22.7 ± 5.1	46 ± 17.2	11 ± 4.5	28 ± 11.7	42 ± 15.6	44 ± 13.0	22 ± 11.0
*Bartelly F1*	81.5 ± 5.4	154 ± 23.1	32 ± 7.0	88 ± 13.2	114 ± 19	120 ± 38.8	72 ± 20.7
*Benarys Gartenfreude*	56.5 ± 5.3	112 ± 30.6	14 ± 4.2	57 ± 20.0	81 ± 23.8	102 ± 72.8	45 ± 15.9
*Primavera*	76.6 ± 6.2	125 ± 19.7	26 ± 6.3	65 ± 11.4	90 ± 13.9	118 ± 48.3	61 ± 16.6
*Black Cherry*	62.8 ± 4.1	96 ± 22.8	19 ± 5.3	59 ± 13.4	68 ± 15.8	91 ± 30.5	49 ± 19.6
*Sakura F1*	66.3 ± 7.2	118 ± 21.6	22 ± 6.3	68 ± 14.2	83 ± 14.5	89 ± 38.3	57 ± 12.6
*Primabella*	59.1 ± 12.5	113 ± 30.8	19 ± 8.8	64 ± 17.1	83 ± 21.6	95 ± 22.2	49 ± 20
*Tastery F1*	71.5 ± 4.2	113 ± 13.8	23 ± 5.5	66 ± 8.0	82 ± 10.9	93 ± 18.2	68 ± 21.2
*Annamay F1*	73.7 ± 12.9	129 ± 26.4	27 ± 6.6	75 ± 16.7	85 ± 18.1	99 ± 32.9	68 ± 8.1
*Amoroso F1*	68.6 ± 10.3	98 ± 18.3	23 ± 3.9	55 ± 11.0	74 ± 10.7	88 ± 32.1	60 ± 23.6
Campari F1	74.4 ± 10.2	121 ± 25.4	24 ± 5.7	68 ± 13.3	85 ± 18.2	93 ± 55.6	52 ± 15.2
Auriga	67.9 ± 8.9	111 ± 17.4	15 ± 3.1	67 ± 9.1	72 ± 10.4	71 ± 26.7	44 ± 20.3
Harzfeuer F1	80.3 ± 10.8	137 ± 21.7	24 ± 5.1	65 ± 9.5	82 ± 14.3	89 ± 35.9	62 ± 16.2
Roterno F1	116.0 ± 7.6	143 ± 40.5	39 ± 13.1	75 ± 21.1	95 ± 28.5	103 ± 31.3	75 ± 18.6
Lyterno F1	110.3 ± 18.5	150 ± 29.3	39 ± 13.6	78 ± 15.2	104 ± 18.4	103 ± 31.8	70 ± 23.1
Bocati F1	112.7 ± 6.7	143 ± 23.3	37 ± 9.1	77 ± 12.8	95 ± 16.8	102 ± 37.1	84 ± 17.7
Cappricia F1	112.1 ± 13.7	155 ± 31.3	43 ± 10.7	71 ± 11.8	103 ± 24.6	98 ± 46.0	76 ± 35.1
Green Zebra	63.9 ± 10.1	110 ± 19.6	22 ± 4.9	70 ± 12.6	75 ± 12.8	76 ± 27.1	47 ± 13.9
Mean	73.9	119	25	66	83	91	58
CV	10.7	17.7	28.6	16.9	18.6	37.1	31.8
HSD	9.9	26.3	8.9	13.9	19.3	42.3	23.2
Year							
2015	70.4 ± 26.1	109 ± 34.4	23 ± 12.0	59 ± 17.1	77 ± 23.5	75 ± 35.9	61 ± 22.5
2016	77.4 ± 23.6	128 ± 33.3	26 ± 10.2	72 ± 17.2	88 ± 22.4	107 ± 38.4	56 ± 24.6

*The nutritional yield is the number of adult males and females who can obtain 100% of the annual recommended dietary allowance from 1 ha of land per year. The cultivars are arranged according to average single fruit weight (see Table 1); cocktail cultivars are indicated in italics; the others are salad cultivars. Mean values are given for each of the 20 cultivars as mean from both years and from each year ± standard deviation. CV, coefficient of variation; HSD, honestly significant difference at p ≤ 0.05.*

The NYs of Ca, K, Mg, and P varied significantly among the cultivars. The variations of NY for the different minerals were as follows: K (46–155 adults year^–1^ ha^–1^), Ca (11–43 adults year^–1^ ha^–1^), Mg (28–88 adults year^–1^ ha^–1^), and P (42–114 adults year^–1^ ha^–1^). As shown in Table 5, “Bartelly F1” was the highest yielding cultivar among cocktail tomatoes and showed the highest NY for all nutrients. “Resi,” known as the lowest yielding cultivar in the present investigation, had also the lowest NY for all the studied minerals.

The salad varieties showed a slightly greater variation in relation to NY. While “Auriga” produced the lowest NY for all the other nutrients with the exception of K (“Green Zebra”) and Mg (“Harzfeuer F1”), the highest NYs were achieved as follows: by “Cappricia F1” for K and Ca, by “Lyterno F1” for Mg, P, and Fe, by “Roterno F1” for Fe, and by “Bocati F1” for Zn. As compared to the NY of macronutrients, higher CV was found for Fe and Zn with values of 37.1 and 31.8%, respectively (Table 5).

Looking at the variation in fruit yield and NY as a function of fruit type, the spectrum of cultivars examined showed different variations in minimum and maximum values. Among the cocktail cultivars, “Resi” had the lowest yield with 22.7 tons ha^–1^, clearly far away from the other cocktail cultivars, while “Bartelly F1” produced a yield 3.5 times higher. This was also reflected in the NYs, where “Resi” produced the lowest NY for all nutrients and “Bartelly F1” the highest; the NYs were 2.7 (P) to 3.3 (K) times higher than in “Resi.” Also, for Fe and Zn, “Bartelly F1” produced 2.7- and 3.3-fold higher NY. For salad cultivars, it was found that, in terms of fruit yield, “Roterno F1” produced about twice as much as “Green Zebra,” the cultivar with the lowest fruit yield. With regard to NY, the differences between the cultivars with the lowest values (“Green Zebra” for K; “Auriga” for Ca, P, Fe, and Zn; “Harzfeuer F1” for Mg) and the highest values (“Cappricia F1” for K and Ca, “Lyterno F1” for Mg and P, “Roterno F1” and “Lyterno F1” for Fe, and “Bocati F1” for Zn) were 0.2 (Mg) to 2.9 (Ca) times higher than those with the lowest values. For the micronutrients Fe and Zn, the maximum values in the above-mentioned cultivars were 0.5 and 2 times higher than in “Auriga.”

### Heritabilities of Traits and Genotypic Values of Cultivars and Their Stability Across Years

The heritabilities and genotypic values of cultivars were calculated for each trait on a single year basis (Tables 6, 7). For the majority of traits such as fruit yield [*H*^2^ = 0.86 (2015), 0.95 (2016)] and densities of K [*H*^2^ = 0.51 (2015), 0.61 (2016)], P [*H*^2^ = 0.54 (2015), 0.74 (2016)], Fe [*H*^2^ = 0.24 (2015), 0.31 (2016)], and Zn [*H*^2^ = 0.21 (2015), 0.43 (2016)], the heritabilities increased in 2016, as expected, since up to four plants were included in the pooled replicates for the traits’ analyses instead of only two plants in 2015. For the traits’ Mg density [*H*^2^ = 0.76 (2015), 0.66 (2016)] and Ca density [*H*^2^ = 0.40 (2015), 0.37 (2016)], the heritabilities remained more or less the same. Traits with the lowest heritabilities were Ca, Fe, and Zn density, respectively.

**TABLE 6:** Genetic parameters [heritability as broad sense heritability (*H*^2^) and genotypic values (best linear unbiased predictors)] of macronutrient densities (Ca, K, Mg, and P) created from a linear fully randomized model on a yearly basis.

	Ca density (mg 100 g^–1^ FW)		K density* (mg 100 g^–1^ FW)		Mg density* (mg 100 g^–1^ FW)		P density (mg 100 g^–1^ FW)	
	2015	2016	2015	2016	2015	2016	2015	2016
H (broad sense)	0.40	0.37	0.51	0.61	0.76	0.66	0.54	0.74
Grand mean (±standard error)	12.07 (±0.66)	12.55 (±0.57)	273.64 (±7.46)	296.18 (±12.16)	11.64 (0.45)	12.97 (0.62)	29.51 (±1.14)	30.99 (±1.77)
Spearman’s rank correlation rho	0.55 *p*-value = 0.01351		0.80 *p*-value = 2.833e-05		0.80 *p*-value = 2.603e-05		0.89 *p*-value < 2.2e-16	
Pearson’s correlation	0.66 *p*-value = 0.001449		0.83 *p*-value = 5.666e-06		0.82 *p*-value = 1.024e-05		0.89 *p*-value = 1.649e-07	

Cultivar = genotype	Geno typic value	Mean (predicted)	Rank	Geno typic value	Mean (predicted)	Rank	Geno typic value	Mean (predicted)	Rank	Geno typic value	Mean (predicted)	Rank	Geno typic value	Mean (predicted)	Rank	Geno typic value	Mean (predicted)	Rank	Geno typic value	Mean (predicted)	Rank	Geno typic value	Mean (predicted)	Rank
Amoroso F1	0.753	12.820	7	−0.574	11.979	13	−28.457	245.183	16	−46.150	250.030	16	−1.287	10.348	15	−2.005	10.968	16	−1.228	28.280	13	−3.523	27.464	15
Annamay F1	1.247	13.313	6	0.377	12.931	7	16.265	289.905	7	9.872	306.052	8	1.453	13.088	7	0.791	13.764	9	−0.204	29.304	10	−1.553	29.434	9
Auriga	−4.035	8.031	20	−3.264	9.290	20	4.639	278.279	11	−11.042	285.138	11	1.002	12.638	8	0.649	13.622	10	−2.392	27.116	15	−3.163	27.824	13
Bartelly F1	2.468	14.534	4	1.346	13.899	3	36.079	309.719	2	33.506	329.686	6	1.812	13.447	3	1.756	14.729	6	4.526	34.034	4	5.777	36.764	4
Benarys Gartenfreude	−3.447	8.620	19	−2.401	10.153	19	8.060	281.700	9	86.933	383.113	1	−1.213	10.423	14	2.822	15.795	3	0.311	29.819	8	10.852	41.839	2
Black Cherry	−1.023	11.043	14	−0.914	11.640	17	−16.916	256.724	15	−23.949	272.231	14	0.136	11.771	11	0.051	13.023	11	−1.495	28.013	14	−3.229	27.758	14
Bocati F1	0.066	12.132	10	−0.749	11.805	16	−44.697	228.943	19	−78.557	217.623	19	−2.130	9.506	17	−4.006	8.967	18	−5.911	23.597	19	−10.233	20.754	19
Campari F1	0.120	12.186	9	−0.605	11.948	14	4.249	277.889	12	−16.367	279.813	13	0.059	11.695	12	−0.588	12.385	12	0.124	29.632	9	−2.083	28.904	10
Cappricia F1	2.496	14.563	3	0.344	12.897	8	−40.778	232.862	17	−49.669	246.511	17	−3.432	8.203	20	−4.108	8.865	19	−5.225	24.283	18	−7.485	23.502	18
Goldita	4.583	16.649	1	1.773	14.327	2	20.635	294.275	6	29.590	325.770	7	1.562	13.198	5	1.623	14.596	7	2.090	31.598	5	1.658	32.645	7
Green Zebra	−0.323	11.743	12	0.165	12.718	9	21.710	295.350	5	−13.052	283.128	12	2.370	14.006	2	1.096	14.068	8	0.986	30.494	6	−2.563	28.424	12
Harzfeuer F1	−2.685	9.382	18	0.642	13.195	6	7.915	281.555	10	9.359	305.539	9	−1.412	10.224	16	−1.609	11.364	15	−3.648	25.860	16	−4.049	26.938	16
Lyterno F1	1.423	13.489	5	−0.530	12.023	12	−42.979	230.661	18	−52.890	243.290	18	−2.605	9.031	18	−3.130	9.843	17	−4.364	25.144	17	−6.814	24.173	17
Primabella	−1.567	10.499	17	0.123	12.676	10	27.919	301.559	3	43.660	339.840	4	1.673	13.308	4	1.979	14.952	5	4.873	34.381	3	5.422	36.409	5
Primavera	−0.950	11.117	13	0.730	13.284	5	−10.619	263.021	14	−0.904	295.276	10	−1.165	10.471	13	−0.936	12.037	14	−1.035	28.473	12	0.486	31.473	8
Resi	3.059	15.126	2	5.828	18.382	1	22.657	296.297	4	78.903	375.083	3	1.557	13.193	6	5.087	18.060	1	9.715	39.223	1	19.999	50.986	1
Roterno F1	0.217	12.284	8	−0.146	12.407	11	−53.413	220.227	20	−80.999	215.181	20	−2.948	8.687	19	−4.157	8.816	20	−6.781	22.727	20	−10.681	20.306	20
Sakura F1	−1.172	10.894	15	0.883	13.436	4	2.471	276.111	13	35.221	331.401	5	0.261	11.896	10	2.146	15.119	4	−0.597	28.911	11	3.530	34.517	6
Supersweet 100 F1	−1.285	10.781	16	−2.297	10.257	18	57.020	330.660	1	81.075	377.255	2	3.600	15.235	1	3.372	16.345	2	9.425	38.933	2	10.182	41.169	3
Tastery F1	0.055	12.122	11	−0.732	11.822	15	8.240	281.880	8	−34.539	261.641	15	0.705	12.341	9	−0.833	12.140	13	0.829	30.337	7	−2.529	28.458	11

The genotypic values were ranked for single years, and correlations between both years were calculated. *Could not estimate Pooled Rep cause of singularity but we kept it in the model. After checking, it did not change the model results.

**TABLE 7:** Genetic parameters [heritability as broad sense heritability (*H*^2^) and genotypic values (best linear unbiased predictors)] of micronutrient densities (Fe and Zn) and fruit yield created from a linear fully randomized model on a yearly basis.

	Fe density (mg 100 g^–1^ FW)		Zn density (mg 100 g^–1^ FW)		Fruit yield (kg/pooled rep)	
	2015	2016	2015	2016	2015	2016
*H* (broad sense)	0.24	0.31	0.21	0.43	0.86	0.95
Grand mean (±standard error)	0.54 (±0.04)	0.70 (±0.05)	0.35 (±0.03)	0.25 (±0.01)	3.52 (±0.29)	3.87 (±0.28)
Spearman’s rank correlation rho	0.52 *p*-value = 0.02098		0.64 *p*-value = 0.002778		0.88 *p*-value < 2.2e-16	
Pearson’s correlation	0.49 *p*-value = 0.02709		0.66 *p*-value = 0.001582		0.95 *p*-value = 2.582e-10	

Cultivar = genotype	Genotypic value	Mean (predicted)	Rank	Genotypic value	Mean (predicted)	Rank	Genotypic value	Mean (predicted)	Rank	Genotypic value	Mean (predicted)	Rank	Genotypic value	Mean (predicted)	Rank	Genotypic value	Mean (predicted)	Rank
Amoroso F1	0.036	0.574	7	−0.050	0.646	13	0.004	0.351	10	−0.020	0.228	13	−0.454	3.065	14	−0.054	3.814	9
Annamay F1	0.000	0.539	10	−0.034	0.662	12	0.099	0.447	4	−0.002	0.246	11	−0.222	3.297	11	0.209	4.077	6
Auriga	−0.097	0.442	18	−0.092	0.604	15	−0.083	0.264	17	−0.025	0.223	15	−0.209	3.310	10	−0.373	3.495	14
Bartelly F1	−0.006	0.533	11	0.100	0.796	4	0.016	0.363	9	0.057	0.305	3	0.357	3.876	6	0.386	4.254	5
Benarys Gartenfreude	−0.014	0.525	12	0.302	0.998	1	0.027	0.374	8	0.086	0.333	1	−0.889	2.630	17	−0.803	3.065	18
Black Cherry	0.006	0.545	9	0.075	0.771	8	−0.008	0.340	11	0.035	0.283	6	−0.408	3.111	12	−0.678	3.190	17
Bocati F1	−0.086	0.452	15	−0.199	0.497	20	−0.088	0.260	18	−0.082	0.166	20	2.192	5.711	1	1.588	5.456	4
Campari F1	−0.083	0.456	14	0.068	0.764	9	−0.071	0.277	16	0.019	0.267	8	0.047	3.566	8	0.003	3.871	8
Cappricia F1	−0.149	0.389	20	−0.168	0.529	17	−0.101	0.247	20	−0.064	0.184	18	1.845	5.364	3	1.879	5.747	3
Goldita	0.094	0.633	3	0.099	0.795	5	0.115	0.463	1	0.017	0.265	9	−1.523	1.996	19	−1.537	2.331	19
Green Zebra	0.028	0.567	8	−0.140	0.556	16	−0.036	0.312	13	−0.052	0.196	16	−0.637	2.882	15	−0.330	3.538	13
Harzfeuer F1	−0.095	0.444	17	−0.021	0.675	11	−0.068	0.280	14	−0.019	0.228	12	0.850	4.369	5	−0.233	3.635	11
Lyterno F1	−0.081	0.458	13	−0.185	0.511	18	−0.069	0.279	15	−0.062	0.186	17	1.511	5.029	4	2.047	5.915	1
Primabella	0.210	0.749	2	0.024	0.720	10	0.039	0.387	6	0.015	0.263	10	−1.008	2.511	18	−0.426	3.442	15
Primavera	0.076	0.615	5	0.076	0.772	7	0.103	0.451	2	0.035	0.283	7	0.125	3.644	7	0.141	4.009	7
Resi	0.222	0.760	1	0.168	0.864	2	0.103	0.451	3	0.050	0.297	4	−2.421	1.098	20	−2.562	1.306	20
Roterno F1	−0.108	0.431	19	−0.193	0.504	19	−0.096	0.252	19	−0.067	0.181	19	2.175	5.694	2	1.929	5.797	2
Sakura F1	−0.095	0.444	16	0.081	0.777	6	−0.009	0.339	12	0.046	0.294	5	−0.409	3.110	13	−0.329	3.539	12
Supersweet 100 F1	0.093	0.632	4	0.156	0.852	3	0.084	0.432	5	0.057	0.305	2	−0.886	2.633	16	−0.660	3.208	16
Tastery F1	0.051	0.589	6	−0.066	0.630	14	0.039	0.387	7	−0.024	0.224	14	−0.035	3.484	9	−0.198	3.670	10

The genotypic values were ranked for single years, and correlations between both years were calculated. *Could not estimate Pooled Rep cause of singularity but we kept it in the model. After checking, it did not change the model results.

To make conclusions about genotypic stability across years, the genotypic values were presented as BLUPs and ranked for single years, and correlations were calculated (Tables 6, 7). High correlations between BLUPs of each year for single traits can be observed, and cultivar ranks do not change much across years. This was observed for fruit yield, K, Mg, and P densities, all having medium-high heritability combined with a strong correlation between genotypic values (fruit yield: 0.88 Spearman’s, 0.95 Pearson; K density: 0.80 Spearman’s, 0.83 Pearson; Mg density: 0.80 Spearman’s, 0.82 Pearson; P density: 0.89 Spearman’s, 0.89 Pearson). The traits Ca density (0.55 Spearman’s, 0.66 Pearson), Fe density (0.52 Spearman’s, 0.49 Pearson), and Zn density (0.64 Spearman’s, 0.66 Pearson) have medium-high correlations, resulting in moderate genotype stabilities since the ranks change likely due to genotype-by-year effects but not completely across years. Ca, Fe, and Zn are also reported with larger CV values than those of the other traits under study (Table 3).

## Discussion

### Nutrient Concentration

Vegetables are an important part of daily diet, and therefore the concentrations of mineral nutrients in them could contribute significantly to the mineral nutrient intake in human diet (Marles, 2017). The present study confirms that mineral concentrations in tomato fruits were strongly influenced by the cultivar (Kapoulas et al., 2013), and the influence of the cultivar on both macro- and micronutrients depends only to some extent on the cultivation year. A high correlation between genotypic values of each year for a single trait showed that, even though there were significant genotype-by-year effects, the cultivar ranks did not change much (Tables 6, 7). A strong correlation of genotypic values across years together with a high heritability of the trait shows that genetics control the trait strongly and genotypes are stable across environments. Thus, the cultivars exhibit a good genotype stability across years for the traits K, Mg, and P density, respectively. The traits Ca density, Fe density, and Zn density exhibited medium-strong correlations and medium heritabilities and therefore resulted in moderate genotype stability. Overall, most cultivars have relatively high genotype stability for different traits across years. It may be that there is a stronger environmental influence on the density of micronutrients (Fe and Zn) than for most of the macronutrients.

Micronutrient density, defined as the amount of a nutrient per unit weight in a food, is important to achieve an optimal micronutrient status in human diet (Miller and Welch, 2013). Basically, the analysis of the mineral concentration of tomatoes in the present study shows that these cultivars can potentially provide essential nutrients to a large population (Table 3). From the present study, data for K, Mg, P, Fe, and Zn concentrations were comparable to those from other studies for organically grown tomatoes (Hernández Suárez et al., 2007; Ordóñez-Santos et al., 2011; Kapoulas et al., 2013; Mohammed et al., 2019). However, the range of the Ca concentration in our tomato cultivars (7.9–17.7 mg 100 g^–1^ FW) was lower than the range of earlier experiments with 28 cultivars (11.2–24.5 mg 100 g^–1^ FW) (Mohammed et al., 2019), but, with the exception of the concentration in one cultivar, it was higher than the range presented by Kapoulas et al. (2013) (8.1–9.0 mg 100 g^–1^ FW) as well as for three tomato cultivars grown in a greenhouse and for five cultivars in open-field cultivation (5.9–7.0 mg 100 g^–1^ FW) (Hernández Suárez et al., 2007). Ca is a mineral with the lowest levels of adequately estimated intake worldwide (Beal et al., 2017).

Potassium is the most abundant mineral nutrient in fresh tomato fruits (Sager, 2017; Labate et al., 2018), while Mg is the mineral nutrient most frequently lacking in the human diet (White and Broadley, 2005). Phosphorus is one of the 17 key elements required in plant metabolism (Dixon et al., 2020) and is essential for human nutrition, but dietary P deficiency occurs very rarely, as it is contained in many foods and is well absorbed (Vorland et al., 2017). High fruit K concentrations (211–361 mg 100 g^–1^ FW), which exceed the range presented by others (Hernández Suárez et al., 2007; Kapoulas et al., 2013; Mohammed et al., 2019; Ordóñez-Santos et al., 2011), were determined in the present study. The achieved result showed a higher maximum content of Mg (16 mg 100 g^–1^ FW) than in other studies (Ordóñez-Santos et al., 2011; Mohammed et al., 2019). The maximum value of P in our tomato cultivars (46.1 mg 100 g^–1^ FW) was slightly higher than those previously reported by Mohammed et al. (2019), who had found P contents up to 43.7 mg 100 g^–1^ FW and far higher than the one presented by Hernández Suárez et al. (2007) (27.1 mg 100 g^–1^ FW). The differences in the range of mineral concentrations reported in different studies may result from variations in the number of the cultivars studied, the type of genetic materials used, the location, and the growing environment evaluated. Regardless of their variation in nutrient concentration, the reductions in the levels of K, Ca, and Mg as well as the increasing concentrations of P and Fe in tomatoes grown in the 1930s and the 1980s [UK Government’s Composition of Foods tables, cited in Mayer (1997)] were reported by Mayer (1997). The author noted that the average concentration of K, Ca, and Mg in tomato fruits decreased from 288 to 250 mg 100 g^–1^ FW, 13.3 to 7 mg 100 g^–1^ FW, and 11 to 7 mg 100 g^–1^ FW, respectively, whereas the concentration of P had witnessed a slight increase from 21 to 24 mg 100 g^–1^ FW over an approximate 50-year period.

Several agrobiodiversity-related studies have focused on improving diets or dietary quality, including intake of key nutrients, dietary diversity, and consumption of micronutrient-rich foods (e.g., Powell et al., 2015; Herrero et al., 2017). From the nutritional perspective, Fe and Zn are essential micronutrients for both humans and plants, but they remain deficient in the diets of the global population (Li et al., 2017). As reported in earlier studies (Ordóñez-Santos et al., 2011; Kapoulas et al., 2013; Mohammed et al., 2019), Fe is identified as a major micronutrient in the tomato fruit. Comparing the micronutrient contents in tomato fruits between the 1930s and 1980s, only Fe was found in higher concentrations in the fruits, with an increase of 16% in tomatoes cultivated 50 years later (Mayer, 1997). In the present study, the range of Fe concentrations was lower than the one measured by Mohammed et al. (2019) and Ordóñez-Santos et al. (2011). On the other hand, the range of the Zn concentration exceeds the range presented by Hernández Suárez et al. (2007); Kapoulas et al. (2013), and Mohammed et al. (2019).

Different values of CV between the traits for the nutrient concentration and also for the fraction of DRI and NY were observed in the present study (Tables 3, 4, and 5). The CV, a mean-standardized measure of variation, is often used to compare the variability of quantitative traits, and higher CVs are ascribed to a greater relative variability (Ogunniyan and Olakojo, 2014; Pélabon et al., 2020). For nutrient concentrations, the highest CV was obtained for Zn followed by Fe (Table 3). The genetic parameters support these findings as Zn and Fe densities exhibit the lowest heritabilities (Table 7). High CVs (Table 3) are related to low heritabilities (Tables 6, 7). These results imply that the concentration of the micronutrients had higher variability than those for macronutrients among the studied parameters. These high CV values of the traits were adequate to distinguish the cultivars. With the CVs between 20 and 30%, Ca had high data dispersion around the mean, thereby reflecting a relatively higher genetic variation (Ene et al., 2016), while K, Mg, and P traits showed the lowest CVs within the range of 10 to 20% (Table 3) and highest heritabilities (Table 6). No CV was lower than 10%—this means that the observed traits in the study displayed moderate to high variability. The variations for CV and the genetic parameters calculated in the present study confirm that the traits were often more controlled by the cultivar (genotype) than by the environment (Kapoulas et al., 2013).

### Contribution of Tomatoes to the DRI of Mineral Nutrients

The contribution of tomato, in terms of macro- and micronutrients, to daily requirements is not very high due to the low dry matter content of the fruit. It was not the primary purpose of this study to evaluate the contribution of the cultivars investigated, but the fraction of DRI was determined in order to calculate the NY. However, the variations in the variety spectrum are also reflected here, so the contribution to the intake of the minerals was rather low, except for K and Fe (Table 4). Our result indicated that 100 g of “Supersweet 100 F1” contributed up to 7.7% of the DRI of K, which agrees with the data reported by Mohammed et al. (2019) after recalculation of the data. Consumption of one serving of tomato (∼200 g) provided 10% of the DRI of K (Labate et al., 2018). Compared to Ca, a greater contribution of Mg and P in the cultivars was observed, with maximal contribution of 100 g fresh fruits up to 4.4 and 6.6% of the DRI reference value for average adult male and female, respectively. These results are important because the intake of minerals, particularly K, Ca, and Mg, in human diets is below healthful levels and often insufficient (Labate et al., 2018).

Interestingly, the cocktail cultivar “Resi” contributed the most to the DRI among the investigated minerals—except for K, thereby showing a valuable performance of this cultivar in terms of nutritional value. In spite of its low fruit productivity, “Resi” has excellent fruit quality and moderate resistance against *Phytophthora infestans* (Zörb et al., 2020).

### Fruit Yield and Its Variation in the Nutritional Yields of the Cultivars

The trait fruit yield exhibits a strong correlation between the genotypic values of both years, and even though significant genotype-by-year effects may appear, the cultivars’ ranks across years do not change much (Table 7). A strong correlation together with a high heritability of fruit yield shows that genetics strongly control the trait. Thus, the cultivars exhibit a high genotype stability for fruit yield across years. The mean fruit yield of the 20 studied cultivars (73.9 ton ha^–1^) exceeded the estimated average yield of global tomato released by FAO (2018), which was about 42 tons ha^–1^ (FAOSTAT, 2020), and higher than the mean of 14 tomato landraces (34.2 ton ha^–1^) reported by Firas et al. (2012). Considering the average yields among all the tested cultivars, “Roterno F1,” “Lyterno F1,” “Bocati F1,” and “Cappricia F1” exhibited the highest fruit yields in both cultivation years (Table 5). These four salad cultivars may represent good materials for tomato production under outdoor organic conditions for fruit yield. “Bartelly F1” showed the highest fruit yield among all cocktail cultivars across the 2 years, and it is, therefore, considered a feasible plant material for cocktail fruit-type tomato production.

With respect to NY, the present study identifies open-field tomato cultivars suitable for organic production with high NYs for the minerals Ca, K, Mg, P, Fe, and Zn (Table 5). The NY incorporates measures of two important dimensions for future food systems: the production of nutritious food and the efficient use of land (De Fries et al., 2015). The present study shows a high variation of the NY of Zn and Fe within the 20 tomato cultivars, thereby indicating a wide variability for these traits in the fruits. Several studies for the organic production of tomatoes (Ordóñez-Santos et al., 2011; Kapoulas et al., 2013; Pavithra et al., 2015) confirm that the genotype has important effects on most of the variations in the micronutrient content (e.g., Fe and Zn), while environmental effects have only a small impact. The metrics of NY opens up options to compare the usefulness of different production systems for food production to feed the growing global population (Moreira-Ascarrunz et al., 2016). Hence, a comparison of the mean average NYs of macronutrients (Ca, K, Mg, and P) and micronutrients (Fe and Zn) of the studied tomato cultivars with the calculated NYs for vegetables under organic production was performed, i.e., for eggplant (Raigón et al., 2010), potato tuber (Hajšlová et al., 2005; Järvan and Edesi, 2009), and tomato (Kapoulas et al., 2013) (Table 8). The means of NY of the eggplant (Raigón et al., 2010) and potato varieties (Järvan and Edesi, 2009) were considerably lower for all the studied nutrients compared to that of the tomato cultivars from the present study (Table 8). The NYs of Fe and Zn calculated from eight potato varieties during 3 years of cultivation (Hajšlová et al., 2005) also only amounted to one third of the NY obtained in the tomato cultivars that we studied. Compared to the study of Kapoulas et al. (2013) with three tomato cultivars, the mean of NYs in the present study showed 1.03-fold higher NY for Fe and 1.66-fold higher NY for Zn. Although the tomato cultivars in the present study had substantially higher NYs for K and Ca, the NYs for Mg and P of the tomato cultivars calculated based on the data in Kapoulas et al. (2013) were 1.70- and 1.52-fold higher than the NY generated from the present study (Table 8). The reasons for the different results are, on the one hand, the different cultivars and, on the other hand, the cultivation conditions. In contrast to our experiment, the trials described in Kapoulas et al. (2013) were carried out under greenhouse conditions and with a higher nutrient supply (including for P). Among different crop species compared from the cited studies, the tomato cultivars that we studied were leading in the NYs for Fe and Zn, and eggplant was the lowest micronutrient-yielding vegetable crop (Table 8).

**TABLE 8:** Nutritional yield (adults ha^–1^ year^–1^) of macro- and micronutrients from different vegetables (organically cultivated) in comparison with the data of the present study.

Study	Species	Year of cultivation	Number of cultivars	Nutritional yield (adults ha^–1^ year^–1^)					
				Ca	K	Mg	P	Fe	Zn
Raigón et al., 2010	Eggplant	2008	3	11	69	29	50	19	26
Hajšlová et al., 2005	Potato	1996–1998	8	–	–	–	–	30	18
Järvan and Edesi, 2009	Potato	2007–2008	2	8	57	46	52	30	18
Kapoulas et al., 2013	Tomato	2008–2010	3	17	66	112	126	88	35
Present study	Tomato	2015–2016	20	25	119	66	83	91	58

*The nutritional yield values of other studies were calculated based on the given data in the cited studies.*

It is notable in the present investigation that the cultivars with the highest yield (“Roterno F1,” “Lyterno F1,” “Bocati F1,” and “Cappricia F1”) were not the cultivars with the highest concentrations (expressed in mg 100 g^–1^ FW) of the six minerals studied. Conversely, the cultivar with the highest mineral concentrations, such as “Resi,” did not show a high yield and was even recorded as the lowest-yielding cultivar. Thus, the results generally show significant antagonism effects of yield trait and mineral concentrations, which means that the positive effect of a high fruit yield is diminished by a decreased nutrient concentration in the fruits. Earlier studies already reported the negative relationship between yield components and mineral concentrations among tomato cultivars (Costa et al., 2011; Chávez-Servia et al., 2018). Davis (2009) reveals inverse relationships between crop yield and mineral concentration, suggesting the presence of the so-called dilution effect, which commonly occurs when selective breeding successfully increases crop yields. The dilution effect of an increased crop yield or harvest index without a proportional increase in the mineral concentration has been well documented in several vegetable and grain crops (Marles, 2017). In this study, we use the metrics of NY which weigh the conventional yield measure (tons/ha) by its nutritional content, and therefore our selected tomato cultivars are supposed to be adequately dense in mineral nutrients to fulfill the requirements with a high yield potential.

The data from the present study shows that 1 ha of land of cultivated “Bartelly F1” can annually produce Mg for 88 adults, P for 114 adults, and Fe for 120 adults. Despite having fewer metric tons per hectare compared to the salad cultivar “Cappricia F1” and other high-yielding cultivars, the cocktail cultivar “Bartelly F1” was excellent in providing Mg, P, and Fe for a high number of people. Nevertheless, “Cappricia F1” was an excellent salad cultivar to supply the dietary intake of Ca, K, and Zn with NYs of 43, 155, and 76 adults year^–1^ ha^–1^, respectively. The determined NY can influence the choice of varieties if both a high fruit yield and a high nutrient density per unit area are to be produced. Moreover, this study provides information about the number of people that can be fed according to their need for different nutrients with the respective tomato cultivars for 1 year from 1 ha of land. In addition, the NY might be an additional metric considered in exploiting the diversity of tomato to satisfy the nutritional needs of an increasing population.

In terms of contribution to the DRI and NY for Fe, the cocktail cultivar “Bartelly F1” produced the highest results, while the salad cultivar “Bocati F1” showed the highest values for Zn. Thus, to gain both maximum agronomic productivity in terms of fruit yield and Fe and Zn NYs, choosing “Bartelly F1” and “Bocati F1” as genotypes for breeding and/or selection in terms of micronutrients would be more feasible. Nevertheless, it should not be interpreted as implying that those cultivars were the sole source of any given nutrient since other cultivars also had significantly high concentrations of Fe and Zn. For example, “Lyterno F1” and “Roterno F1” did not differ significantly from “Bartelly F1” in the NY of Fe, and “Cappricia F1” and “Roterno F1” did not vary significantly with “Bocati F1” for the NY of Zn. However, if we compare the cultivars within the group of fruit type, it is clear that, among cocktail tomatoes, “Bartelly F1” produced the highest NY for both Fe and Zn. Incidentally, while comparing within the salad tomatoes, “Bocati F1” would be the best choice because it had the highest NY for both Fe and Zn.

Across the 2-year investigation, our data indicate that the cocktail cultivar “Resi” accounted for three- to fourfold lower NY values as compared to the highest-performing salad cultivars of the respective determined nutrients—for instance, the NY for Zn of “Bocati F1” (84 adults year^–1^ ha^–1^) was about fourfold higher than that measured in “Resi” (22 adults year^–1^ ha^–1^). Therefore, it might be possible to increase the mineral concentrations in tomatoes by incorporating the micronutrient-dense cultivars in breeding programs that have a higher ability to accumulate the Fe and Zn contents in the fruits. Even though the NY covers both yield and nutrient concentration, it should not imply that a single crop be consumed as the sole source of any given nutrient (Graham et al., 2018) as monotonous diets are likely to increase the risk of various nutrient deficiencies (Arimond et al., 2010). The NY should rather be considered as a policy tool to emphasize how any number of agronomic decisions are implicitly biased against either yield or nutrient concentration (Graham et al., 2018).

The current study reveals that a cultivar with the highest fruit yield was not automatically the cultivar with the highest NY. However, high yielding-cultivars with considerable nutrient contents were likely to have higher NYs. Similarly, the cultivar with the highest nutrient content did not always show a high NY. The results show that it is a challenge to evaluate genotypes for breeding and/or selection in terms of overall nutritional quality (Moreira-Ascarrunz et al., 2016; Gascuel et al., 2017) because no single cultivar might be rich in all relevant compounds or nutrients (Vicente et al., 2009), and no parent is a good general combiner for all desirable traits (Agarwal et al., 2017). Nonetheless, the metric of NY could be useful for plant breeders and growers in the selection, development, and choice of cultivars, and on the global scale, it provides another perspective for policymakers in projections of food demand and requirements of nutrients (De Fries et al., 2015). The results of this study regarding DRI and NY, together with the high genotypic stability of several cultivars for yield and micro- and macronutrients across years, provide valuable input to growers’ and plant breeders’ decisions. However, consumer demand will still determine the growers’ choice on tomato cultivars and influence the plant breeders’ cultivar development program.

The NYs of the micronutrients mentioned in the present study were much higher and satisfying in comparison with the NYs of tomatoes and other vegetables calculated from previous reports. In fact, the comparison with other plant species should consider the effects of diverse cultivation systems. Data for both mineral contents and crop yields are also lacking in many studies. The results show that the tomato biodiversity used in the present study can contribute to satisfying the nutritional needs of the ever-growing human population while minimizing the negative impact on the environment. Hence, the metrics of NY can also be useful in selecting the cultivar with improved nutrients to increase food security and conserve the biodiversity of tomato in organic outdoor production.

## Data Availability Statement

The original contributions presented in the study are included in the article/Supplementary Material, further inquiries can be directed to the corresponding author.

## Author Contributions

CE, MN, and EP planned and designed the experimental setup. CE performed the experiments. CE and SG analyzed the data. All the authors wrote the manuscript.

## Conflict of Interest

The authors declare that the research was conducted in the absence of any commercial or financial relationships that could be construed as a potential conflict of interest.
