# Supplementary material for: Biodiversity in Tomatoes: Is It Reflected in Nutrient Density and Nutritional Yields Under Organic Outdoor Production?
Source: Front Plant Sci. 2020 Nov 24;11:589692. doi: 10.3389/fpls.2020.589692 (PMC7732668; doi:10.3389/fpls.2020.589692)
Supplement: Supplementary file 1 [file Data_Sheet_1.pdf]

SUPPLEMENTARY MATERIAL

Table S1. Dietary reference intake (DRI) for adult males and females (not pregnant or lactating) aged between 19 and 50 (Institute of Medicine, 2006)

**Nutrients (mg day^{-1})**	**Male 19-30 y**	**Female 19-30 y**	**Male 31-50 y**	**Female 31-50 y**	**Average**
Ca ǂ	1,000	1,000	1,000	1,000	1,000
K ǂǂ	4,700	4,700	4,700	4,700	4,700
Mg ǂ	400	310	420	320	363
P ǂ	700	700	700	700	700
Fe ǂ	8	18	8	18	13
Zn ǂ	11	8	11	8	9.5

ǂ: RDA Recommended dietary allowance: defined as the average daily dietary intake level sufficient to meet the nutrient requirement of nearly all (97–98%) healthy individuals in a particular life-stage and gender group (Murphy and Poos, 2002)

ǂǂ: AI Adequate intake: defined as a recommended intake level based on observed or experimentally determined approximations or estimates of nutrient intake by a group (or groups) of healthy people that are assumed to be adequate – used when an RDA cannot be determined (Murphy and Poos, 2002)

y- years

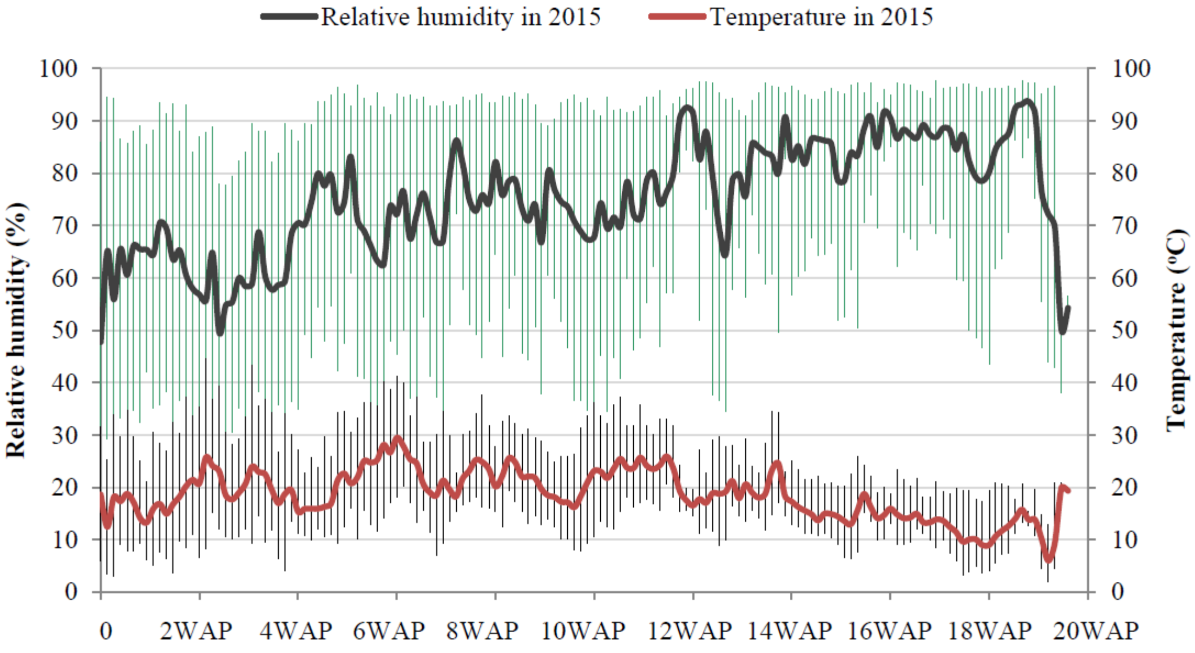

Figure S1.1 Average daily temperature and relative humidity during the experiment in 2015

Error bars present daily minimum (lower side) and maximum (upper side) of the relative humidity and temperature. Data were recorded by using an EBI 20-TH Data Logger (ebro Electronic GmbH & Co. KG). The device recorded temperature and relative humidity every 30 minutes; daily mean was derived from 48 data points.

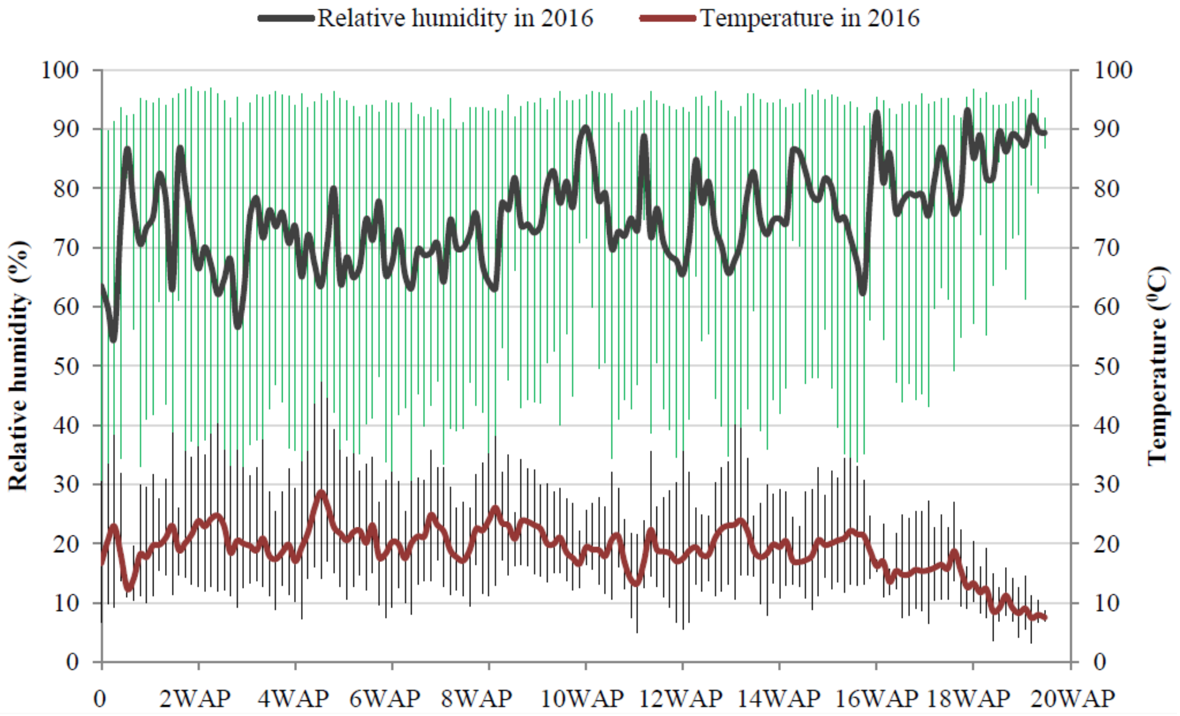

Figure S1.2 Average daily temperature and relative humidity during the experiment in 2016

Error bars present daily minimum (lower side) and maximum (upper side) of the relative humidity and temperature. Data were recorded by using an EBI 20-TH Data Logger (ebro Electronic GmbH & Co. KG). The device recorded temperature and relative humidity every 30 minutes; daily mean was derived from 48 data points.
